# Bidirectional structural-functional connectivity coupling reorganization in obstructive sleep apnea

**DOI:** 10.3389/fnins.2026.1890168

**Published:** 2026-08-10

**Authors:** Baoting Chao, Gang Han, Xueqing Chen, Jie Chen

**Affiliations:** 1 Department of Radiology, Shandong Provincial Hospital Affiliated to Shandong First Medical University, Jinan, Shandong, China; 2 Department of Radiology, Weifang Traditional Chinese Hospital, Weifang, Shandong, China; 3 Shandong Second Medical University, Weifang, Shandong, China; 4 Department of Interventional Radiology, Shandong Provincial Hospital Affiliated to Shandong First Medical University, Jinan, Shandong, China

**Keywords:** hypoxia, multimodal MRI, obstructive sleep apnea, structural-functional coupling, vigilance

## Abstract

**Background:**

Obstructive sleep apnea (OSA) is associated with widespread cognitive and neural dysfunction; however, how chronic intermittent hypoxia and sleep fragmentation reshape the relationship between structural and functional brain connectivity remains unclear. Structural–functional connectivity (SC-FC) coupling provides a multimodal framework for characterizing the correspondence between anatomical architecture and functional communication.

**Methods:**

Fifty patients with moderate-to-severe OSA and fifty matched healthy controls (HCs) underwent overnight polysomnography and multimodal MRI scanning, including resting-state functional MRI and diffusion tensor imaging. Regional SC-FC coupling was calculated using SC and FC matrices derived from the Brainnetome Atlas. Group differences in regional SC-FC coupling were examined across 246 regions, and exploratory correlation analyses were performed with cognitive and clinical measures.

**Results:**

Compared with HCs, patients with OSA showed significantly increased SC-FC coupling in frontal-insular, posterior default-mode, sensorimotor, and visual regions. In contrast, significantly decreased SC-FC coupling was observed in temporal-associative, limbic, and subcortical regions. Increased SC-FC coupling in the superior frontal gyrus, precuneus, and insula was associated with poorer psychomotor vigilance performance, including prolonged reaction times and increased lapse frequency. Reduced hippocampal and subcortical coupling was associated with nocturnal hypoxia burden, including increased percentage of sleep time spent below 90% oxygen saturation and lower minimum oxygen saturation. Inferior parietal lobule coupling showed a weaker association with apnea-hypopnea index severity.

**Conclusion:**

OSA is associated with bidirectional regional SC-FC coupling reorganization, characterized by hypercoupling in attention-related systems and hypocoupling in limbic-subcortical regions. These exploratory findings suggest that distinct coupling patterns may relate to different clinical dimensions of OSA, including vigilance impairment and nocturnal hypoxic burden.

## Introduction

Obstructive sleep apnea (OSA) is a prevalent sleep-related breathing disorder characterized by recurrent upper airway collapse during sleep, leading to intermittent hypoxia, sleep fragmentation, and repeated physiological stress (Patel, 2019; Tasali et al., 2025). Although OSA is primarily known for its respiratory consequences, it has become increasingly recognized for its broader impact on brain function and behavior (Chu et al., 2026; Zhang et al., 2025). Cognitive dysfunction, particularly in areas related to attention and vigilance, is among the most frequently reported symptoms in OSA patients (Mazza et al., 2005; Olaithe et al., 2018; Rosenzweig et al., 2016). This evidence suggests that OSA is not merely a nocturnal disorder, but a chronic condition with significant daytime brain function impairment.

Neuroimaging studies have consistently shown that OSA is associated with structural and functional abnormalities in a variety of brain regions (Wu et al., 2025). These include the frontal cortex, insula, precuneus/posterior cingulate, hippocampus, thalamus, parietal cortex, and occipito-temporal areas, as well as key brain networks such as the default mode, salience, executive-attention, and visual networks (Tahmasian et al., 2016; Canessa et al., 2011). These findings support the notion that the neural consequences of OSA are not confined to a specific brain region, but are widespread, involving both cortical and subcortical systems. However, the patterns of alterations are heterogeneous, with some regions exhibiting structural or functional increases, while others show decreases in connectivity or activity (Canessa et al., 2011; Redline et al., 2023).

However, most previous OSA neuroimaging studies have examined structural connectivity (SC) and functional connectivity (FC) separately. Such a single-modality perspective may be insufficient to clarify how chronic OSA-related pathology reshapes the relationship between brain anatomy and brain function. Brain structure and function are inherently coupled, and the degree of correspondence between SC and FC may itself provide important information about disease-related brain organization (Fotiadis et al., 2024). In this context, SC-FC coupling offers a multimodal framework for quantifying the relationship between anatomical pathways and functional communication, thereby extending beyond conventional structural-only or functional-only analyses (Honey et al., 2009; Suárez et al., 2020).

This framework may be particularly relevant for OSA. Because OSA involves chronic intermittent hypoxia and repeated sleep disruption, its neural effects are unlikely to be uniform across the brain. Instead, different systems may show distinct forms of structure–function reorganization. In some regions, functional communication may become more tightly constrained by anatomy, whereas in others the correspondence between structure and function may weaken. Such region-specific alterations may help explain the heterogeneous patterns reported in prior OSA imaging studies and provide a more integrated account of OSA-related brain abnormalities. Recent work using regional SC-FC coupling has shown that coupling alterations can reveal disease-related brain reorganization that is not fully captured by structural or functional measures alone (Xu et al., 2024; Liu et al., 2026). Importantly, increased coupling may reflect stronger anatomical tethering of function, whereas decreased coupling may indicate relative structure–function decoupling.

Therefore, the present study investigated regional SC-FC coupling alterations in patients with OSA using a multimodal framework integrating structural and resting-state FC. We aimed to determine whether OSA is associated with region-specific reconfiguration of SC-FC coupling and to explore the potential clinical relevance of these alterations. Importantly, altered SC-FC coupling does not necessarily indicate a unidirectional effect. Increased coupling may reflect stronger anatomical tethering of functional communication and reduced functional flexibility, whereas decreased coupling may indicate relative structure–function decoupling and impaired integrative processing. Therefore, both stronger and weaker coupling may be maladaptive, albeit in different ways, and may show distinct associations with clinical severity and cognitive manifestations in OSA.

## Methods

### Participants and clinical assessment

A total of 60 patients with suspected moderate-to-severe OSA and 60 healthy controls (HCs) were enrolled in this study. The OSA and HC groups were matched for age, sex, and years of education. OSA diagnosis was confirmed using overnight polysomnography (PSG) according to the criteria of the American Academy of Sleep Medicine (Berry et al., 2012). Only patients with an Apnea-Hypopnea Index (AHI) ≥ 15 events/h were included in the OSA group. All patients with OSA were newly diagnosed and untreated at the time of MRI assessment. None had received continuous positive airway pressure therapy, oral appliance therapy, upper-airway surgery, or other OSA-specific treatment before enrollment. HCs underwent PSG examination to exclude sleep-disordered breathing and were required to have an AHI < 5 events/h.

Participants were excluded if they had: (1) neurological disorders, including stroke, epilepsy, or neurodegenerative disease; (2) major psychiatric disorders; (3) severe cardiovascular disease; (4) alcohol or substance abuse; (5) other sleep disorders; or (6) contraindications to MRI scanning. Participants with excessive head motion during MRI acquisition (>1.5 mm translation or >1.5° rotation) were also excluded from further analysis.

Clinical and sleep-related measures, including body mass index (BMI), AHI, oxygen desaturation index (ODI), mean and minimum oxygen saturation (SaO₂), arousal index, Epworth Sleepiness Scale (ESS), Pittsburgh Sleep Quality Index (PSQI), Montreal Cognitive Assessment (MoCA), and percentage of sleep time spent below 90% oxygen saturation (Ts90), were collected for all participants.

The study was approved by the Ethics Committee of Shandong Provincial Hospital Affiliated to Shandong First Medical University, and written informed consent was obtained from all participants prior to enrollment.

### Cognitive assessment

Sustained attention and vigilance were assessed using the Psychomotor Vigilance Task (PVT). Participants were seated comfortably in a quiet room and instructed to respond as quickly as possible to visual stimuli appearing on a computer screen. The task lasted 10 min, during which reaction times (RTs) and lapses (responses >500 ms) were recorded. Mean RT, fastest RT, slowest RT, and number of lapses were calculated for each participant. The PVT has been validated as a sensitive measure of attentional performance under sleep fragmentation and intermittent hypoxia conditions (Xu et al., 2025). All participants completed the PVT on the same day as MRI scanning to ensure temporal proximity between behavioral and neuroimaging assessments. The PVT data were then used in correlation analyses with regional SC-FC coupling measures.

### Polysomnography

All participants underwent overnight polysomnography (PSG) in the sleep laboratory using a standard clinical monitoring system. Physiological recordings included electroencephalography (EEG), electrooculography (EOG), electromyography (EMG), electrocardiography (ECG), airflow monitoring, thoracoabdominal respiratory effort, and pulse oximetry. Sleep stages and respiratory events were scored according to the guidelines of the American Academy of Sleep Medicine. Apnea was defined as a ≥ 90% reduction in airflow lasting at least 10 s, and hypopnea was defined as a ≥ 30% reduction in airflow accompanied by oxygen desaturation or arousal. The Apnea-Hypopnea Index (AHI) was calculated as the number of apnea and hypopnea events per hour of sleep. Additional PSG-derived parameters included the oxygen desaturation index (ODI), arousal index, mean oxygen saturation, minimum oxygen saturation, and percentage of sleep time spent below 90% oxygen saturation (Ts90).

### MRI acquisition

MRI data were collected using a 3 T GE MR750 scanner equipped with a standard 8-channel head coil at the Department of Radiology, Shandong Provincial Hospital Affiliated to Shandong First Medical University. Resting-state functional MRI images were acquired using a single-shot gradient-echo echo-planar imaging (EPI) sequence with the following parameters: repetition time (TR) = 2000 ms, echo time (TE) = 30 ms, flip angle = 90°, field of view (FOV) = 240 × 240 mm^2^, matrix size = 64 × 64, slice thickness = 3.5 mm without interslice gap, and 45 axial slices covering the whole brain. A total of 240 volumes were acquired for each participant. During scanning, participants were instructed to keep their eyes closed, remain awake, avoid systematic thinking, and minimize head motion throughout the scan. High-resolution T1-weighted anatomical images were acquired using a three-dimensional spoiled gradient recalled echo sequence with the following parameters: repetition time (TR) = 8.2 ms, echo time (TE) = 3.18 ms, flip angle = 9°, field of view (FOV) = 256 × 256 mm^2^, matrix size = 512 × 512, slice thickness = 1 mm, and 196 sagittal slices. Diffusion tensor imaging (DTI) data were acquired using a single-shot spin-echo echo-planar imaging sequence with the following parameters: repetition time (TR) = 8,500 ms, echo time (TE) = 85 ms, field of view (FOV) = 256 × 256 mm^2^, matrix size = 128 × 128, slice thickness = 2 mm without interslice gap, 70 axial slices, diffusion weighting factor (b-value) = 1,000 s/mm^2^, and diffusion sensitizing gradients applied along 64 non-collinear directions, along with one non-diffusion-weighted (b0) image. The functional, structural, and diffusion MRI data were subsequently preprocessed for SC-FC coupling analysis as described below.

### Data preprocessing

All imaging data were preprocessed and analyzed using established neuroimaging software packages and custom scripts. Resting-state fMRI data were preprocessed using the default preprocessing pipeline implemented in the CONN functional connectivity toolbox version 20b running in MATLAB R2022 (Whitfield-Gabrieli and Nieto-Castanon, 2012). The preprocessing steps involved several stages: exclusion of the first five volumes to allow for T1 equilibrium, correction for head motion using rigid-body transformation, slice timing correction to align the acquisition times of the slices, and scrubbing for artifact removal using a method based on framewise displacement (FD). The data were then normalized to Montreal Neurological Institute (MNI) space, resampling the data to a 2 × 2 × 2 mm^3^ voxel size, followed by spatial smoothing with a 6 mm full-width at half-maximum (FWHM) Gaussian kernel. Participants whose translation or rotation parameters exceeded ±1.5 mm or ±1.5° at any time point were excluded from further analysis. T1-weighted images were segmented into gray matter, white matter, and cerebrospinal fluid to support spatial normalization and voxel-wise denoising. The time series of each voxel was further denoised by linear detrending, regression of nuisance signals, and temporal filtering. Nuisance regressors included the Friston 24-parameter head-motion model, mean white-matter signal, mean cerebrospinal-fluid signal, and detected outlier volumes. A temporal band-pass filter of 0.01–0.10 Hz was applied to reduce low-frequency drift and high-frequency physiological noise.

DTI data were preprocessed using FSL version 6.0.5. The preprocessing procedure included extraction of the b0 image, brain extraction to remove non-brain tissue, correction for head motion and eddy-current distortions, and diffusion tensor estimation. Fractional anisotropy maps were generated for quality control and registration purposes. Deterministic tractography was performed using FSL, and the resulting streamlines were used to construct participant-specific SC matrices.

### FC matrix construction

FC was estimated using the preprocessed resting-state fMRI data. Regions of interest were defined using the Brainnetome Atlas (Fan et al., 2016), which parcellates the brain into 246 cortical and subcortical regions across both hemispheres. For each participant, the mean time series of each region was calculated by averaging the time series of all voxels within that region. Pairwise FC between all region pairs was computed using Pearson correlation coefficients. These correlation coefficients were Fisher z-transformed and arranged into a 246 × 246 FC matrix for each participant.

### SC matrix construction

SC was estimated from the preprocessed diffusion MRI data using deterministic tractography in FSL version 6.0.5. The same Brainnetome 246-region atlas was used to define the nodes of the structural network, thereby ensuring correspondence between the structural and functional connectivity matrices. For each participant, the number of reconstructed streamlines connecting each pair of regions was calculated. To reduce the influence of potentially spurious connections, fiber counts below the 25th percentile of the participant-specific nonzero fiber-count distribution were set to zero. The SC edge weight between each pair of regions was then calculated as the fiber count normalized by the mean volume of the two connected regions. This procedure yielded a 246 × 246 SC matrix for each participant.

### SC-FC coupling

Regional SC-FC coupling was calculated using a multimodal predictive modeling framework. First, the original SC matrix was transformed into 35 additional SC-derived weighted matrices using the Brain Connectivity Toolbox and custom MATLAB scripts. These matrices were designed to characterize complementary structural communication strategies, including both centralized and decentralized communication processes. Specifically, the derived matrices included six shortest path length matrices, consisting of one binary matrix and five weighted matrices with gamma = 0.12, 0.25, 0.50, 1.00, and 2.00; two communicability matrices, including one binary and one weighted matrix; two cosine similarity matrices; six search information matrices; six path transitivity matrices; two matching index matrices; four navigation matrices, including two number-of-hops and two total-distance matrices; two mean first passage time matrices; six flow graph matrices, including three binary and three weighted matrices with Markov time t = 1, 2.5, and 5; and one Euclidean distance matrix based on the MNI coordinates of the Brainnetome regions. Together with the original SC matrix, this procedure generated 36 SC-weighted matrices for each participant.

Principal component analysis was then applied to the 36 vectorized SC-weighted matrices to obtain orthogonal anatomical predictors. For each participant, the smallest number of principal components explaining more than 80% of the total variance was retained. Across participants, this procedure retained a median of 6 components, with a range of 5–8 components, explaining 83.6 ± 2.4% of the total variance. These retained components were used as predictors in a multilinear regression model to estimate the FC profile of each brain region.

For a given region i, the empirical FC profile was defined as the vector of Fisher z-transformed FC values between region i and all other regions. The corresponding SC-derived predictor profiles were obtained from the retained principal component matrices. A multilinear regression model was fitted as follows:


$$FC_{i}=\beta_{0}+\sum_{k=1}^{n}\beta_{k}PC_{k,i}+\varepsilon_{i}$$

where $FC_{i}$ represents the empirical functional connectivity profile of region *i*; $PC_{k,i}$ represents the kth structural connectivity-derived principal component predictor profile; $\beta_{k}$ represents the regression coefficient; and $\varepsilon_{i}$ represents the residual error. The predicted FC profile was then correlated with the empirical FC profile using Pearson correlation. This correlation coefficient was defined as the regional SC-FC coupling value for region *i*. Thus, one SC-FC coupling value was obtained for each of the 246 Brainnetome regions in each participant. The obtained correlation coefficients were Fisher z-transformed before group-level statistical analyses.

**Figure 1:**
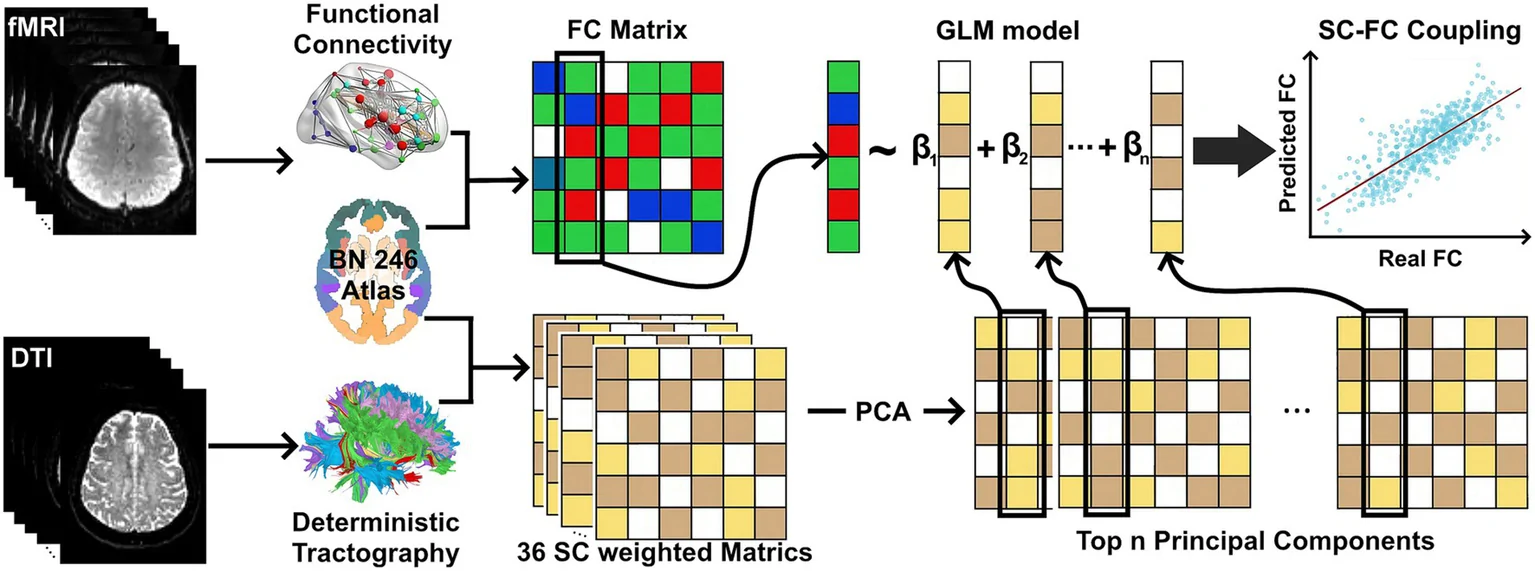
Schematic of SC-FC coupling calculation. Resting-state functional MRI and DTI data were acquired for each participant. FC matrices were derived from fMRI time series using the Brainnetome 246-region atlas. SC matrices were computed from deterministic tractography of DTI data. Multiple SC-weighted matrices were then reduced using PCA to extract the top components representing dominant structural patterns. The retained principal components explained more than 80% of the variance in the SC-derived communication features and were used to predict regional FC profiles. A general linear model (GLM) was applied to predict FC from SC principal components, and the predicted FC was compared with the observed FC to calculate SC-FC coupling. The resulting SC-FC coupling reflects the degree to which functional connectivity is constrained by underlying anatomical connectivity across brain regions.

This approach quantified SC-FC coupling at the regional rather than network level and reflected the extent to which the FC profile of each brain region could be predicted from its SC-derived communication profile. The workflow for SC-FC coupling calculation is illustrated in Figure 1.

### Statistical analysis

Statistical analyses were performed in MATLAB using custom scripts. The normality of continuous variables was assessed using the Shapiro–Wilk test and visual inspection of distributional plots. Normally distributed variables are presented as mean ± standard deviation, whereas non-normally distributed variables are presented as median and interquartile range. Categorical variables are presented as counts and percentages. Demographic, clinical, polysomnographic, and cognitive variables were compared between the OSA and HC groups using independent-sample *t*-tests or Mann–Whitney U tests for continuous variables, as appropriate according to data distribution. Categorical variables were compared using the chi-square test or Fisher’s exact test. Group differences in regional SC-FC coupling were tested separately for each of the 246 Brainnetome regions using two-sample *t*-tests or nonparametric tests, as appropriate according to data distribution. To correct for multiple regional comparisons, *p* values from the 246 regional tests were adjusted using the Benjamini–Hochberg false-discovery-rate procedure. Statistical significance for regional group comparisons was defined as an FDR-adjusted q value < 0.05.

Associations between altered regional SC-FC coupling and clinical or cognitive measures were examined as exploratory analyses in the OSA group. Correlation analyses were focused on regions showing significant between-group differences in SC-FC coupling. Pearson correlation coefficients were used for normally distributed variables, and Spearman rank correlation coefficients were used for non-normally distributed variables. To account for multiple testing, correlation *p* values were adjusted using the Benjamini–Hochberg false-discovery-rate procedure across the six reported association tests. FDR-adjusted *p* values are reported for the correlation analyses.

Sensitivity partial-correlation analyses were additionally performed to evaluate whether the observed associations were robust after controlling for age, sex, years of education, and body mass index. Because polysomnographic severity and hypoxemia measures, including AHI, ODI, Ts90, and oxygen saturation indices, are highly interrelated, models simultaneously including multiple severity indices were not used as the primary analysis to avoid multicollinearity and overadjustment. All statistical tests were two-sided. Statistical significance was set at *p* < 0.05 for uncorrected demographic comparisons and FDR-adjusted *p* < 0.05 for regional and correlation analyses requiring multiple-comparison correction.

## Results

### Participant characteristics

Of the 60 initially enrolled patients with suspected moderate-to-severe OSA, 10 were excluded before the final analysis: 5 did not meet the PSG criterion for moderate-to-severe OSA, 2 had incomplete clinical or imaging data, and 3 were excluded because of excessive head motion or insufficient fMRI or DTI data quality. Among the 60 initially enrolled potential HCs, 10 were excluded: 3 showed PSG evidence of sleep-disordered breathing, 4 had incomplete clinical or imaging data, and 3 were excluded because of excessive head motion or insufficient fMRI or DTI data quality. Therefore, 50 patients with moderate-to-severe OSA and 50 HCs were included in the final analysis. No significant group differences were observed in age, sex distribution, or years of education (all *p* > 0.05). Compared with HCs, OSA patients exhibited significantly higher body mass index (BMI), apnea-hypopnea index (AHI), oxygen desaturation index (ODI), arousal index, and percentage of sleep time spent below 90% oxygen saturation (Ts90) (all *p* < 0.001). In addition, OSA patients showed significantly reduced mean and minimum oxygen saturation levels during sleep (both *p* < 0.001).

Sleep architecture analysis revealed significantly increased N1 sleep proportion and significantly reduced N3 and REM sleep proportions in the OSA group compared with HCs (all *p* < 0.001), whereas no significant difference was observed for N2 sleep proportion (*p* > 0.05). OSA patients also demonstrated significantly higher Epworth Sleepiness Scale (ESS) and Pittsburgh Sleep Quality Index (PSQI) scores, along with significantly lower Montreal Cognitive Assessment (MoCA) scores (all *p* < 0.001).

Detailed demographic, clinical, and polysomnographic characteristics are summarized in Table 1.

**Table 1:** Demographic and clinical characteristics of participants.

Variables	HC (*n* = 50)	OSA (*n* = 50)	Statistic	*P* value
Age (years)	41.2 ± 8.5	43.8 ± 9.1	t = −1.42	0.159
Sex (M/F)	28/22	31/19	χ^2^ = 0.36	0.548
Education (years)	15.1 ± 2.8	14.7 ± 3.1	t = 0.68	0.499
BMI (kg/m^2^)	23.4 ± 2.1	30.4 ± 3.8	t = −11.50	<0.001
ESS	4.8 ± 2.5	10.1 ± 4.0	t = −7.90	<0.001
PSQI	4.2 ± 1.8	8.3 ± 3.0	t = −8.30	<0.001
PSG parameters				
AHI (/h)	2.3 ± 1.4	42.5 ± 18.2	Z = −8.72	<0.001
ODI (/h)	1.7 ± 1.2	33.4 ± 16.5	Z = −8.55	<0.001
Mean SaO₂ (%)	96.1 ± 0.9	93.5 ± 1.8	t = 9.20	<0.001
Minimum SaO₂ (%)	90.2 ± 2.4	76.4 ± 8.7	t = 10.80	<0.001
Ts90 (%)	0.3 ± 0.5	14.8 ± 10.3	Z = −8.61	<0.001
N1 sleep (%)	7.6 ± 2.8	22.0 ± 7.3	t = −12.60	<0.001
N2 sleep (%)	51.7 ± 6.1	52.3 ± 7.4	t = −0.44	0.662
N3 sleep (%)	19.0 ± 5.8	10.5 ± 5.3	t = 7.70	<0.001
REM sleep (%)	21.8 ± 4.6	15.2 ± 5.1	t = 6.80	<0.001
Arousal index (/h)	8.4 ± 3.1	37.8 ± 14.5	Z = −8.40	<0.001
MoCA	27.6 ± 1.3	25.9 ± 2.0	t = 5.00	<0.001
PVT performances				
Mean RT (ms)	350.2 ± 38.2	421.1 ± 121.9	Z = −4.62	<0.001
Fastest RT (ms)	286.7 ± 22.9	290.2 ± 30.9	t = −0.64	0.523
Slowest RT (ms)	426.4 ± 51.8	637.7 ± 166.4	Z = −5.70	<0.001
Number of lapses	2.4 ± 1.3	8.4 ± 5.2	Z = −6.35	<0.001

Data are presented as mean ± standard deviation. Independent-samples *t*-tests were used for normally distributed variables. Mann–Whitney U tests were used for non-normally distributed variables. Chi-square tests were used for categorical variables. OSA, obstructive sleep apnea; HC, healthy controls; AHI, apnea–hypopnea index; ODI, oxygen desaturation index; ESS, Epworth Sleepiness Scale; PSQI, Pittsburgh Sleep Quality Index; PSG, polysomnography; Ts90, percentage of sleep time spent with oxygen saturation below 90%; MoCA, Montreal Cognitive Assessment; PVT, Psychomotor Vigilance Task; RT, reaction time.

### Cognitive performance

Compared with HCs, patients with OSA demonstrated significantly impaired psychomotor vigilance performance. Specifically, the OSA group exhibited significantly prolonged reaction times (RTs) and increased numbers of lapses during the PVT (both *p* < 0.001). In addition, OSA patients demonstrated significantly lower MoCA scores compared with HCs (*p* < 0.001). Cognitive performances are summarized in Table 1.

### Group differences in SC-FC coupling

Significant regional alterations in SC-FC coupling were observed in patients with OSA compared with HCs.

Compared with HCs, OSA patients exhibited significantly increased SC-FC coupling in the superior frontal gyrus (SFG), middle frontal gyrus (MFG), orbital frontal gyrus (OrG), precuneus, middle temporal gyrus (MTG), insula, postcentral gyrus (PostCG), occipital gyrus (OcG), superior occipital gyrus (sOcG), and cuneus (all FDR-adjusted q < 0.05). These regions were predominantly distributed within frontal, salience-related, sensorimotor, and occipital cortical systems.

In contrast, significantly decreased SC-FC coupling was observed in the inferior temporal gyrus (ITG), fusiform gyrus, inferior parietal lobule (IPL), hippocampus, and subcortical regions in the OSA group relative to HCs (all FDR-adjusted q < 0.05). These regions were mainly located within temporal–parietal associative, limbic, and subcortical systems.

**Figure 2:**
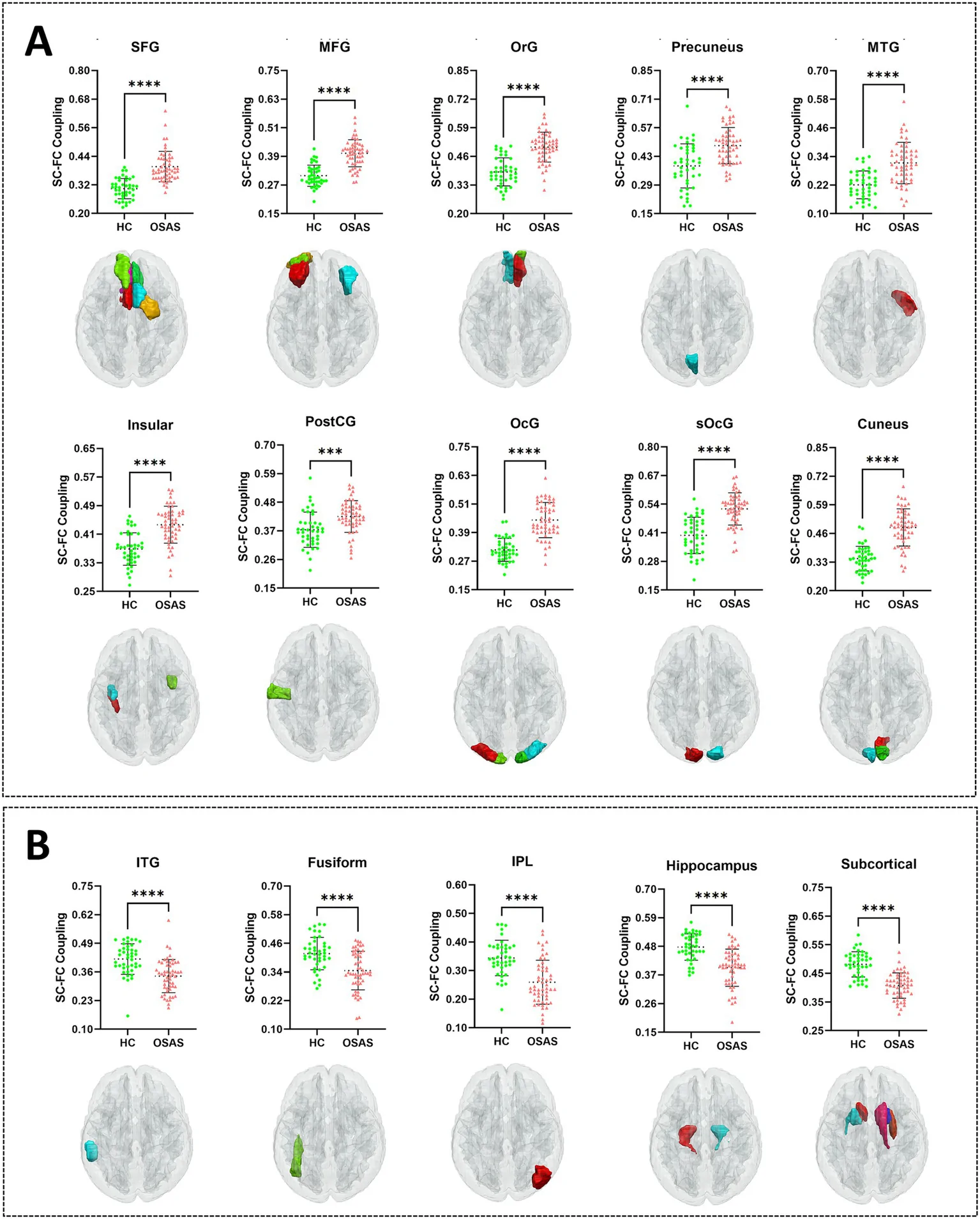
Bidirectional alterations in regional SC-FC coupling in OSA. **(A)** Hypercoupling regions: Scatter plots and corresponding 3D renderings of superior frontal gyrus (SFG), middle frontal gyrus (MFG), orbital frontal gyrus (OrG), precuneus, middle temporal gyrus (MTG), insula, postcentral gyrus (PostCG), occipital gyrus (OcG), superior occipital gyrus (sOcG), and cuneus. OSA patients showed significantly increased SC-FC coupling relative to HCs (all FDR-adjusted *q* < 0.05). **(B)** Hypocoupling regions: Scatter plots and corresponding 3D renderings of inferior temporal gyrus (ITG), fusiform gyrus, inferior parietal lobule (IPL), hippocampus, and subcortical regions. OSA patients showed significantly decreased SC-FC coupling relative to HCs (all FDR-adjusted q < 0.05). Each scatter plot shows individual participant values with mean ± SD, and each 3D rendering illustrates the anatomical location of the corresponding region on a transparent cortical surface. Significance levels are indicated as follows: ****FDR-adjusted *q* < 0.01; ***FDR-adjusted *q* < 0.05.

The spatial distribution of SC-FC coupling alterations demonstrated distinct regional patterns across cortical and subcortical brain systems. Detailed statistical results are presented in Figure 2.

### Correlation analyses

**Figure 3:**
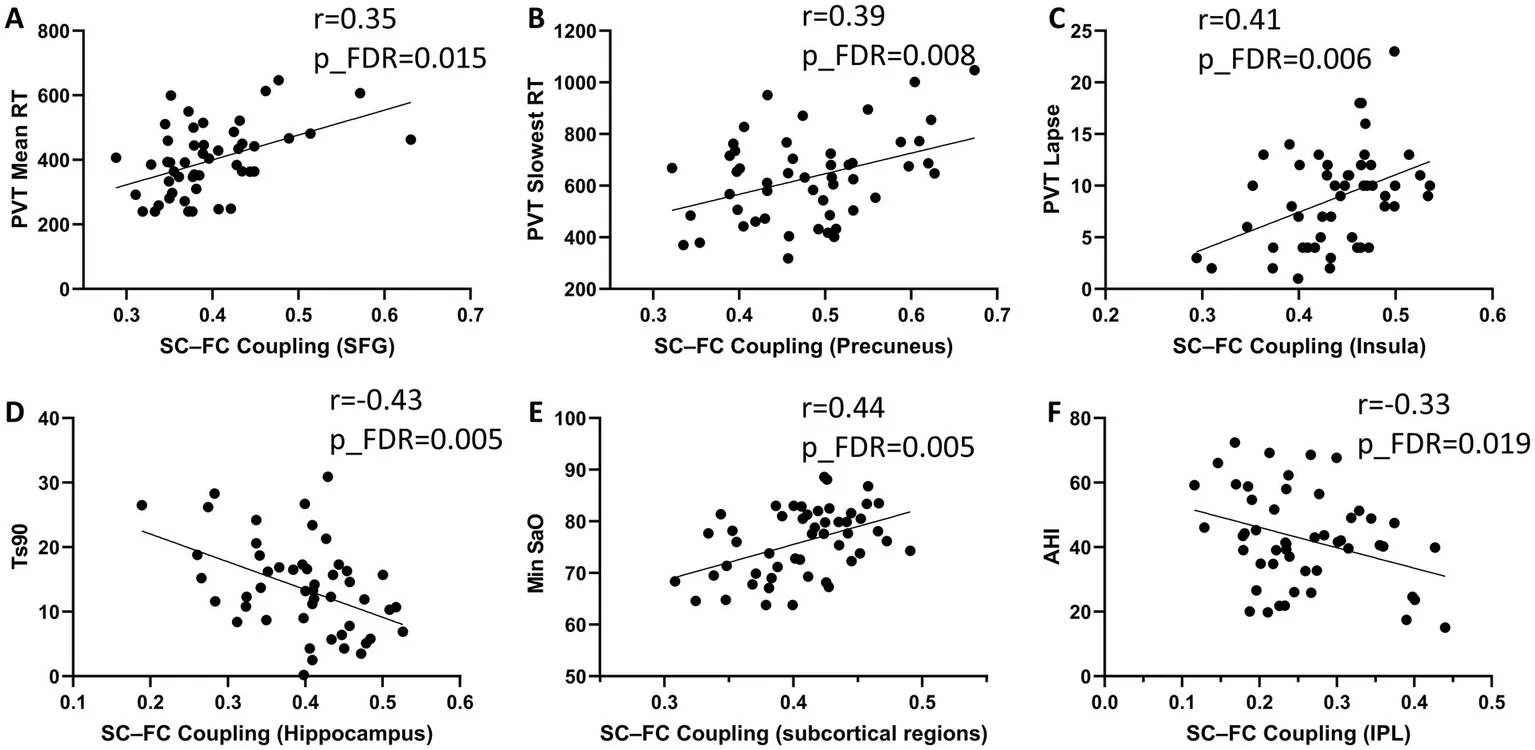
Exploratory correlations between regional SC-FC coupling and cognitive/clinical measures in patients with OSA. Scatter plots illustrate exploratory associations between regional SC-FC coupling and cognitive or clinical measures in patients with OSA. **(A)** Positive correlation between SC-FC coupling in the superior frontal gyrus (SFG) and mean reaction time (RT) on the Psychomotor Vigilance Task (PVT). **(B)** Positive correlation between SC-FC coupling in the precuneus and slowest PVT RT. **(C)** Positive correlation between SC-FC coupling in the insula and number of lapses during the PVT. **(D)** Negative correlation between hippocampal SC-FC coupling and percentage of sleep time spent below 90% oxygen saturation (Ts90). **(E)** Positive correlation between SC-FC coupling in subcortical regions and minimum oxygen saturation (Min SaO₂). **(F)** Negative correlation between SC-FC coupling in the inferior parietal lobule (IPL) and apnea-hypopnea index (AHI). Each point represents an individual participant. Correlation coefficients and FDR-adjusted *p* values are indicated for each panel. These exploratory associations suggest that hypercoupling and hypocoupling patterns may relate to different clinical dimensions of OSA, including psychomotor vigilance impairment and nocturnal hypoxia burden.

Exploratory correlation analyses were performed to examine the relationships between altered regional SC-FC coupling and cognitive or clinical measures in patients with OSA (Figure 3).

Increased SC-FC coupling in attention-related regions was associated with poorer psychomotor vigilance performance. SC-FC coupling in the SFG was positively associated with mean PVT reaction time (*r* = 0.35, FDR-adjusted *p* = 0.015). SC-FC coupling in the precuneus was positively associated with slowest PVT reaction time (*r* = 0.39, FDR-adjusted *p* = 0.008), and SC-FC coupling in the insula was positively associated with PVT lapse frequency (*r* = 0.41, FDR-adjusted *p* = 0.006).

Reduced SC-FC coupling in limbic and subcortical regions was associated with nocturnal hypoxemia. Hippocampal SC-FC coupling was negatively associated with Ts90 (*r* = −0.43, FDR-adjusted *p* = 0.005), whereas SC-FC coupling in subcortical regions was positively associated with minimum oxygen saturation (*r* = 0.44, FDR-adjusted *p* = 0.005). In addition, IPL coupling was negatively associated with AHI (*r* = −0.33, FDR-adjusted *p* = 0.019).

Sensitivity partial-correlation analyses controlling for age, sex, years of education, and body mass index yielded similar results. The associations between SFG, precuneus, and insula coupling and PVT performance remained in the same direction and were only slightly attenuated. Similarly, hippocampal and subcortical coupling remained associated with hypoxemia-related measures. The association between IPL coupling and AHI was weakened after covariate adjustment and became marginal, suggesting that this finding should be interpreted cautiously. Detailed sensitivity analyses are provided in Supplementary Table S1.

## Discussion

The present study investigated regional SC-FC coupling alterations in patients with moderate-to-severe OSA using a multimodal neuroimaging framework integrating SC and resting-state FC. Our findings revealed widespread but bidirectional alterations in SC-FC coupling in OSA. Specifically, increased SC-FC coupling was mainly observed in frontal-insular, posterior default mode, sensorimotor, and visual cortical regions, including the superior frontal gyrus, middle frontal gyrus, insula, precuneus, postcentral gyrus, occipital cortex, and cuneus. In contrast, decreased SC-FC coupling was primarily distributed in temporal-associative, limbic, parietal, and subcortical regions, including the inferior temporal gyrus, fusiform gyrus, inferior parietal lobule, hippocampus, and subcortical areas.

Importantly, these coupling abnormalities showed distinct association patterns with clinical and cognitive measures. SC-FC coupling in the SFG, precuneus, and insula was associated with poorer PVT performance, including longer mean reaction time, slower reaction time, and increased lapse frequency. By contrast, coupling abnormalities in the hippocampus and subcortical regions were associated with nocturnal hypoxia-related indices, including Ts90 and minimum oxygen saturation. AHI showed a more restricted relationship with SC-FC coupling, with significance observed only in the IPL. These exploratory findings suggest that OSA-related brain reorganization may not be fully captured by a single disease-severity index, but may instead reflect multiple partially dissociable pathological dimensions.

One major finding of the present study was increased SC-FC coupling in frontal-insular and posterior cortical regions that are closely involved in attention regulation and vigilance maintenance. The SFG is implicated in executive control, top-down attentional regulation, and sustained task engagement (Seeley et al., 2007). The insula is a key node of the salience network and contributes to interoceptive monitoring, salience detection, and switching between internally and externally oriented brain states (Menon and Uddin, 2010). The precuneus, as a central hub of the posterior default mode network, is involved in self-referential processing, attentional state regulation, and fluctuations in arousal (Buckner et al., 2008). Attention and vigilance are supported by coordinated interactions among distributed executive-control, salience, and default-mode systems rather than by isolated regional activity (Corbetta and Shulman, 2002). Efficient maintenance of vigilance requires flexible communication among these large-scale networks, allowing adaptive transitions between internally oriented processing and externally driven attentional demands. Therefore, increased SC-FC coupling in the SFG, insula, and precuneus may indicate that functional communication becomes more tightly constrained by underlying structural pathways in OSA, reflecting reduced flexibility of structural-functional communication (Menon and Uddin, 2010). In OSA, repeated sleep fragmentation, micro-arousals, and intermittent hypoxia may disturb adaptive network coordination, resulting in a more rigid relationship between anatomical pathways and functional communication during wakefulness. Therefore, increased coupling in the SFG, insula, and precuneus may represent candidate neural correlates of impaired vigilance regulation, although this interpretation remains exploratory.

In contrast to the hypercoupling pattern, decreased SC-FC coupling was observed in the hippocampus, IPL, ITG, fusiform gyrus, and subcortical regions. These areas are involved in memory processing, multimodal integration, visual-associative processing, and limbic-subcortical regulation. The hippocampus is particularly vulnerable to intermittent hypoxia because of its high metabolic demand and sensitivity to oxidative stress (Bartsch and Wulff, 2015). Reduced SC-FC coupling in the hippocampus may therefore reflect disruption of the normal relationship between anatomical connectivity and functional communication in a hypoxia-sensitive memory-related structure. The correlation results further support this interpretation. Hippocampal SC-FC coupling was negatively correlated with Ts90, indicating that patients who spent a greater proportion of sleep time below 90% oxygen saturation showed lower hippocampal coupling. Similarly, SC-FC coupling in subcortical regions was positively correlated with minimum oxygen saturation, indicating that lower nocturnal oxygen saturation was associated with reduced coupling in these regions. Together, these findings suggest a possible association between nocturnal hypoxia burden and structure–function decoupling in limbic and subcortical systems (Lavie, 2015; Rosenzweig et al., 2014). The divergent coupling directions observed within temporal regions should not necessarily be interpreted as inconsistent. The temporal lobe contains functionally heterogeneous systems, and the MTG itself can be subdivided into distinct subregions with different anatomical and FC profiles (Xu et al., 2015). Previous studies have shown that the MTG participates in multimodal semantic processing, semantic control, and higher-order associative integration (Davey et al., 2016). By contrast, the ITG, fusiform gyrus, and hippocampus are more closely related to ventral visual-associative processing, visual working memory, and associative memory retrieval (Ranganath et al., 2004). Therefore, hypercoupling in the MTG and hypocoupling in other temporal-limbic regions may reflect region-specific reorganization within functionally distinct temporal subsystems rather than a contradictory finding.

The IPL showed a distinct pattern in the present study. The IPL is a higher-order association region involved in attentional reorienting, sensorimotor integration, and multimodal information processing (Decroix et al., 2020). Altered SC-FC coupling in the IPL may therefore be relevant to the attentional and cognitive instability frequently observed in OSA. In the present study, IPL coupling was the only coupling measure associated with AHI, suggesting that respiratory-event frequency may relate to coupling disruption in specific associative cortical regions. However, this association was weaker after covariate adjustment and should be interpreted cautiously. AHI remains the standard index for diagnosing and classifying OSA severity, but it primarily reflects the frequency of respiratory events and does not fully capture the depth, duration, or cumulative physiological burden of nocturnal oxygen desaturation (Azarbarzin et al., 2019; Pinilla et al., 2023). In this sample, hypoxia-related measures showed broader exploratory associations with coupling abnormalities than AHI. This pattern raises the possibility that nocturnal oxygen burden may be more closely related to regional SC-FC coupling alterations than respiratory-event frequency alone (Rosenzweig et al., 2014). Therefore, incorporating hypoxia indices such as Ts90 and minimum oxygen saturation may provide a more informative characterization of OSA-related neural injury.

Taken together, the present findings support a dual-pattern model of SC-FC coupling reorganization in OSA. On one hand, hypercoupling in frontal-insular and precuneus regions was associated with vigilance impairment, suggesting altered structure–function organization within attention-related systems. On the other hand, hypocoupling in hippocampal and subcortical regions was associated with nocturnal hypoxia burden, suggesting hypoxia-related structural-functional decoupling. These apparently inconsistent findings may reflect the fact that different brain systems are affected by different pathological components of OSA. Sleep fragmentation and vigilance instability may preferentially affect frontal-insular attention systems, whereas nocturnal hypoxemia may preferentially affect limbic and subcortical systems. SC-FC coupling provides a useful framework for integrating these effects because it captures the relationship between anatomical architecture and functional communication.

Several limitations should be considered. First, the cross-sectional design limits causal interpretation. Longitudinal studies are needed to determine whether altered SC-FC coupling precedes cognitive decline, emerges as a consequence of chronic OSA exposure, or changes after effective treatment such as continuous positive airway pressure. Second, the sample size was modest for a multimodal neuroimaging study involving regional SC-FC coupling and exploratory clinical association analyses. Although the reported associations were corrected for multiple testing and showed similar directions in covariate-adjusted sensitivity analyses, the correlation coefficients were small to moderate. These findings should therefore be interpreted as exploratory brain–behavior and brain–physiology associations rather than definitive evidence of causal mechanisms. Because multiple regions and clinical outcomes were examined, residual risk of false-positive findings remains possible despite FDR correction. Replication in larger independent cohorts is needed. Third, SC was reconstructed using conventional DTI-based deterministic tractography. This approach provides an indirect approximation of white-matter architecture and may be limited in regions with crossing fibers or complex microstructural organization. Future studies incorporating advanced diffusion models, such as diffusion kurtosis imaging, neurite orientation dispersion and density imaging, or multi-shell diffusion MRI, may improve the characterization of OSA-related microstructural injury and further refine estimates of SC-FC coupling. Fourth, genetic susceptibility was not assessed in the present study. Inherited factors may contribute to interindividual differences in OSA risk, vulnerability to intermittent hypoxia and sleep fragmentation, and related neural injury. Future studies incorporating genetic or polygenic risk information may help explain heterogeneity in SC-FC coupling alterations and clinical manifestations. Fifth, the present study focused on regional SC-FC coupling. Future studies incorporating network-level, dynamic, and longitudinal coupling analyses may further clarify how structural-functional alterations interact across distributed brain systems and whether these alterations are reversible after treatment.

## Conclusion

OSA was associated with bidirectional regional SC-FC coupling reorganization, characterized by increased coupling in frontal-insular and posterior attention-related regions and decreased coupling in limbic-subcortical regions. Exploratory association analyses suggested that hypercoupling was related to poorer psychomotor vigilance performance, whereas hypocoupling was related to greater nocturnal hypoxic burden. Regional SC-FC coupling may provide a promising multimodal framework for characterizing cognitive and hypoxia-related brain alterations in OSA, although larger longitudinal studies are needed to validate its robustness and clinical utility.

## Data Availability

The raw data supporting the conclusions of this article will be made available by the authors, without undue reservation.
