# Supplementary material for: Bidirectional structural-functional connectivity coupling reorganization in obstructive sleep apnea
Source: Front Neurosci. 2026 Aug 10;20:1890168. doi: 10.3389/fnins.2026.1890168 (PMC13500753; doi:10.3389/fnins.2026.1890168)
Supplement: Supplementary file 1 [file Supplementary_file_1.docx]

**Supplementary Table S1. Sensitivity partial-correlation analyses for associations between altered regional SC-FC coupling and clinical or cognitive measures in patients with OSA**

| **Region** | **Direction of group difference** | **Clinical/cognitive variable** | **Primary correlation r** | **Primary raw p** | **Primary FDR-adjusted p** | **Partial r (age, sex, education, BMI)** | **Partial raw p** | **Partial FDR-adjusted p** |
| --- | --- | --- | --- | --- | --- | --- | --- | --- |
| Superior frontal gyrus (SFG) | Increased SC-FC coupling in OSA | Mean PVT reaction time | 0.35 | 0.0125 | 0.015 | 0.32 | 0.030 | 0.036 |
| Precuneus | Increased SC-FC coupling in OSA | Slowest PVT reaction time | 0.39 | 0.0053 | 0.008 | 0.36 | 0.014 | 0.021 |
| Insula | Increased SC-FC coupling in OSA | PVT lapses | 0.41 | 0.0030 | 0.006 | 0.38 | 0.009 | 0.018 |
| Hippocampus | Decreased SC-FC coupling in OSA | Ts90 | -0.43 | 0.0017 | 0.005 | -0.40 | 0.006 | 0.018 |
| Subcortical regions | Decreased SC-FC coupling in OSA | Minimum SaO₂ | 0.44 | 0.0008 | 0.005 | 0.41 | 0.005 | 0.018 |
| Inferior parietal lobule (IPL) | Decreased SC-FC coupling in OSA | Apnea-hypopnea index | -0.33 | 0.0190 | 0.019 | -0.29 | 0.051 | 0.051 |

**Abbreviations:** SC-FC, structural-functional connectivity; OSA, obstructive sleep apnea; PVT, Psychomotor Vigilance Task; Ts90, percentage of total sleep time spent with oxygen saturation below 90%; SaO₂, oxygen saturation; FDR, false discovery rate.

**Notes:** Primary correlations reflect unadjusted correlation analyses in the OSA group. Partial correlations control for age, sex, years of education, and body mass index. FDR-adjusted p values were calculated across the six reported association tests using the Benjamini-Hochberg procedure. The IPL-AHI association was the weakest and became marginal after covariate adjustment, supporting cautious interpretation of AHI-related findings.
